# Structured implementation of digital, systematically updated guideline recommendations for enhanced adherence in schizophrenia (SISYPHOS)—protocol of a cluster-randomized trial

**DOI:** 10.1186/s13063-022-06749-0

**Published:** 2022-09-24

**Authors:** Carolin Lorenz, Gabriele Gaigl, Duygu Güler, Theresa Halms, Naiiri Khorikian-Ghazari, Astrid Röh, Marco Schneider, Elias Wagner, Thomas Schneider-Axmann, Angelika Kapfhammer, Marisa Flick, Charline Pielenz, Eva Salveridou-Hof, Peter Falkai, Wolfgang Gaebel, Alkomiet Hasan, Stefan Leucht

**Affiliations:** 1 Department of Psychiatry and Psychotherapy, Technische Universität München, Klinikum rechts der Isar, Ismaninger Str. 22, 81675 Munich, Germany; 2 Department of Psychiatry, Psychotherapy and Psychosomatics, Medical Faculty, University of Augsburg, Augsburg, Germany; 3 Department of Psychiatry and Psychotherapy, University Hospital, LMU Munich, Munich, Germany; 4 Department of Psychiatry and Psychotherapy, Medical Faculty, LVR-Klinikum Düsseldorf, Heinrich-Heine-University; WHO Collaborating Centre in Quality Assurance and Empowerment in Mental Health DEU-131, Düsseldorf, Germany

**Keywords:** Guideline, Implementation, Schizophrenia, Living guideline, MAGICapp, Cluster-randomized controlled trial

## Abstract

**Background:**

Despite high acceptance rates in the field, the implementation of the 2019 published German evidence and consensus-based S3 guideline is unsatisfactory. This study aims to assess the superiority of an adaptive online version with a better visualization of the recommendations in terms of guideline conformity, application of shared decision making, and digital health expertise compared to the classic pdf print version of the guideline.

**Methods:**

The study is a multicenter, controlled, cluster-randomized trial with two arms: one arm investigating the implementation of the German schizophrenia guideline in form of a digital format (intervention group using the evidence ecosystem MAGICapp), the other arm in form of the classic print pdf version (control group). Physicians and psychologists working in specialized hospitals will be included in the study. The guideline-knowledge before and after the intervention is defined as primary outcome measure. Secondary endpoints include digital health expertise and application of shared decision making.

**Discussion:**

This is the first study evaluating if an adaptive-digital version of the schizophrenia guideline is superior to the classic pdf print version. Therefore, the guideline is digitally prepared in the evidence-ecosystem MAGICapp, which covers the whole process of the development of a living guideline. We intend to use the results of the cluster-randomized trial for developing the German S3 guideline for schizophrenia in form of a living guideline in future.

**Trial registration:**

The study is registered (10 May 2022) in the German Clinical Trials Register (DRKS) and the WHO International Clinical Trials Registry Platform (ICTRP) under registration number DRKS00028895.

**Supplementary Information:**

The online version contains supplementary material available at 10.1186/s13063-022-06749-0.

## Background

Guidelines are nowadays a standard of evidence-based medicine [1–3]. However, the implementation in clinical practice remains in many cases insufficient [4–7]. While the development of clinical practice guidelines has been standardized and improved over the last years, the implementation to improve clinical practice remains challenging [8, 9].

In 2019, the German evidence and consensus-based S3 guideline for schizophrenia was published. A recent study has shown that in spite of a high acceptance of this guideline, more than half of the participants did not use the guideline in everyday clinical practice [10]. In that regard, the question arises of identifying and understanding the reasons for this gap between acceptance and implementation.

Possible reasons for poor guideline adherence include several existing barriers (e.g., too long versions, inappropriate form of publication, lack of familiarity concerning the use of guidelines) and not yet comprehensively considered facilitators (e.g., workshops for practitioners about the content of the guideline) [10–12]. Several frameworks aiming to explain insufficient guideline adherence and to categorize the barriers and facilitators of guideline implementation are available [13, 14]. Moreover, guidelines are often already out of date when published due to the exponentially increasing medical knowledge [15, 16]. One potential solution of this problem is the development of so-called living guidelines. In brief, living guidelines optimize the guideline development process by updating individual recommendations as soon as relevant new evidence becomes available or at least once a year [17, 18]. To ensure such a continuous updating of guidelines, digital, internet and platform-independent based concepts of guideline development are required. One such system is the evidence ecosystem called MAGICapp [19], which covers the whole process of developing living guidelines and includes the possibility of publishing “conventional” guidelines. MAGICapp was chosen as according to the ‘Guidelines International Network Tech-Working Group’ this system seems to be superior to other platforms in terms of functionality [20]. Moreover, the Working Group of the Scientific Medical Societies (AWMF) in Germany plans to use this evidence ecosystem in the future [21]. Further important advantages of MAGICapp (e.g., compared to GRADEpro) are the graphical depiction of the evidence in order to support shared decision-making and the interactive potential of the platform due to the comment function allowing users of the MAGICapp browser-based software to give feedback to each recommendation.

A living guideline for schizophrenia in Germany is currently under development and this study aims to examine whether such an optimized online version improves guideline implementation compared to classic implementation strategies.

This cluster-RCT will be the first study to evaluate the effect of a guideline implementation using the evidence-ecosystem MAGICapp within a circumscribed model region in South Germany involving all major hospitals that are involved in the care of people with schizophrenia. Here, we present the concept, design and protocol of this study.

## Methods/design

### Study objectives

This study is part of a project called ‘*Structured implementation of digital, systematically updated guideline recommendations for enhanced adherence in schizophrenia’* (‘SISYPHOS’) [22]. The major part of the project investigates in form of a cluster-randomized controlled trial if an online version with a visualization of the recommendations is superior in terms of guideline conformity, application of shared decision making, and digital health expertise than the classic pdf print version. Therefore, the current German schizophrenia guideline is digitally prepared in the evidence ecosystem MAGICapp [19]. The following protocol presents the design of the cluster-randomized controlled trial. The protocol was prepared in accordance with the SPIRIT reporting guidelines [23] (see Additional file 1).

### Randomization

The sequence of randomization and the randomization list of the clusters will be performed by a statistician experienced in the method using the Mersenne-Twister algorithm. Randomization is balanced according to the number of clusters (see below) and the structure of the hospitals (university hospitals, large community hospitals, small community hospitals). All statistical analyses will be conducted blindedly as the statistician performing the analyses has no knowledge of which clinic and cluster belong together. A statistical analyses plan will be developed before closing the database.

### Study design

This study is a multicenter, controlled, cluster-randomized, two-arm trial with both arms investigating the implementation of the German schizophrenia guideline. One arm (intervention) in form of an adaptive digital format and the other arm (control) in form of the classic pdf print version. A cluster randomization was chosen in order to avoid discussions between the participants of one organizational unit and thus contamination, which would have been likely to occur with an individual randomization [24]. The trial has been approved in ethical and legal terms by the local ethics committee of the medical faculty of the LMU, Munich (reference number 21-0780) and the involved data protection officer (reference number 1819.b.).

The study involves 17 clinics for psychiatry and psychotherapy in the regions of Swabia and Upper Bavaria, Germany. As these clinics have more than 13,000 employees and provide care for about three million inhabitants, they are the main supplier for people with schizophrenia in South Bavaria. Those 17 clinics are allocated to 14 clusters (see Table 2). A stakeholder of a TUM-located self-aid group is involved in the SISYPHOS Project.

### Study population

Participants have to fulfill the following inclusion criteria: they have to be at least 18 years old (female, male, divers), and they have to be employed by one of the 17 participating clinics as a physician or psychologist. Sufficient knowledge of the German language (defined by the ability to work on patients) is also required.

### Recruitment procedure and study flow

**Fig. 1:**
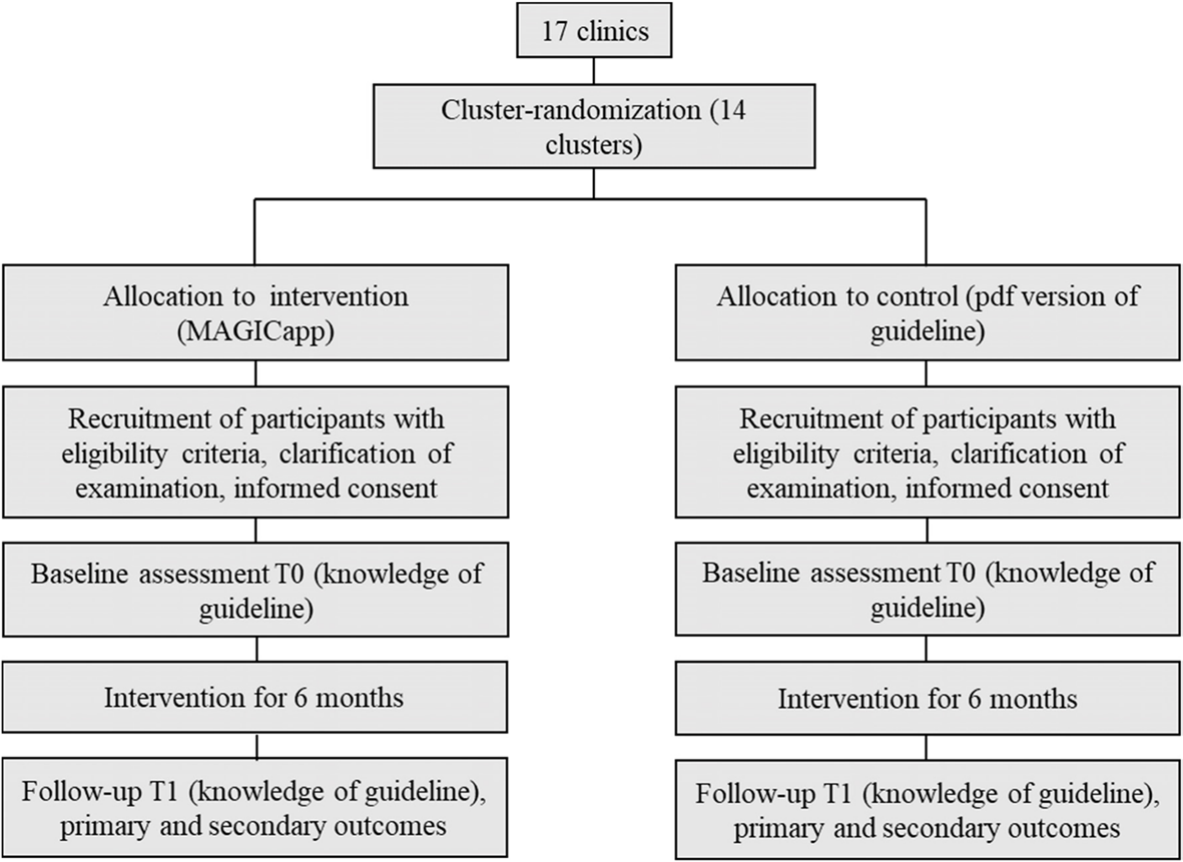
Study design

The recruitment and data collection will be conducted from March 2022 until November 2022. Participants fulfilling the inclusion criteria and having signed informed consent start with the baseline survey (T0). In detail, recruitment and obtaining informed consent was locally performed by the respective hospital directors who were authorized by the responsible ethical board. Signed ICs will be sent to the study coordinator Prof. Hasan. Participation was only possible upon presentation of the signed consent form. After receiving the signed IC, the leading study center provides the link for the baseline survey. When the link was opened, participants will be again asked to confirm their participation and were informed about all data protection standards. The team of the University of Augsburg (Prof. Alkomiet Hasan) coordinated the whole process. They advertise for participation in the study via emails and video conferences, sent the links for the survey, and perform the trainings. In more detail, for the baseline assessment an email is sent with a link to the questionnaire and in addition a pseudonymization code as a replacement for their names using a licensed software LimeSurvey version 5.3.4+ of the LMU Munich. First, a sociodemographic questionnaire comprising 12 questions collects information including, e.g., gender, age, school years, and working experience. Second, we enquire the attitude towards the guideline for schizophrenia as well as a living guideline and the knowledge of the German S3 guideline for schizophrenia (see details below). Afterwards, the intervention follows, either in form of the experimental condition (MAGICapp) or in form of the control condition (print/pdf version) depending on the randomization. Six months later, the intervention stops and the participants are asked to fill in the same questionnaire as at the beginning of the survey (T1) (see Fig. 1, Table 1).

**Table 1:** Overview of examinations at different time points

Time point	Screening	T0	Intervention 6M	T1
Allocation clinics	X			
Inclusion/exclusion criteria	X			
Informed consent	X			
Sociodemographic questionnaire		X		
Questionnaire on guideline knowledge of the S3 Guideline Schizophrenia (primary endpoint)		X		X
eHealth literacy scale		X		X
System Usability Scale		X		X
Provider Decision Process Assessment Instrument		X		X
Participatory Decision Making Questionnaire (PEF-FB-Doc)		X		X
Recording of attitudes and use of the respective formats (traditional print format or MAGICapp)		X		X

### Intervention

In both study arms, a structured, multimodal implementation of the schizophrenia guideline is conducted as follows:

- There are interactive presentations with regard to evidence-based medicine and S3 guidelines. Theoretical knowledge about guidelines will be imparted such as strength of recommendations, the development of guidelines, and the general structure of guidelines as well as the structure of the S3 schizophrenia guideline and practical knowledge how to use the guideline for clinical questions. The slides of the presentation will be provided to the participants. This part will take about 90 min, and due to the SarsCov2-pandemic, it will be performed in a digital format via a version of zoom, licensed and released by the University of Augsburg. Non-interactive recordings of this presentation are offered for participants who are prevented from attending the dates offered.
- All participants are informed that a free pdf version of the long and short version of the S3 guideline schizophrenia is available on the AWMF’s and DGPPN’s (German Association for Psychiatry, Psychotherapy and Psychosomatics) website as most participants are aware of the existence of the guideline in this format. Thus, with all participants receiving this information, a systematic bias factor will be reduced.
- A digital SISYPHOS expert board will take place during the intervention every other week for 60 min, and all participants are invited to take part. This makes a lively exchange possible and current questions as well as difficulties in the treatment of patients with schizophrenia can be discussed and based on concrete questions the expert board explains the recommendation of the guideline in this individual case. The SISYPHOS expert team includes experts of the guideline (Prof. Alkomiet Hasan and Prof. Stefan Leucht) and clinically experienced practitioners. The discussed cases and recommendations as well as the power-point slides will be sent to all participants in written form after each board meeting. This is supposed to serve as a multiplier of the implementation process.
- All participants receive emails during the intervention as a reminder to deal with the schizophrenia guideline.
- Furthermore, the participants are being motivated to use the guideline for shared decision making with their patients in clinical routine.

Clusters are either randomized to the experimental (interventional) group (MAGICapp) or the control group (classic pdf print version) (see Table 2 for clinics and corresponding cluster). In the experimental group, the participating clinics receive a teaching including the concepts, the structure, and the aims of a digital, evidence-based ecosystem such as MAGICapp as well as an interactive hands-on training on how to use MAGICapp. The participants get an access to MAGICapp and have the possibility via the comment function to give feedback to each recommendation and visualization. The control group receives the same teaching but an interactive hands-on training on how to use the classic pdf print version of the guideline for schizophrenia.

**Table 2:** Seventeen clinics and the 14 corresponding clusters

Clinic	Cluster
Klinik für Psychiatrie und Psychotherapie, LMU Klinikum^a^	Cluster 1
Klinik für Psychiatrie und Psychotherapie, TU München^a^	Cluster 2
BKH Augsburg, University of Augsburg^ab^	Cluster 3
MPI für Psychiatrie^a^	Cluster 4
kbo Klinik Wasserburg^b^	Cluster 5
kbo Klinik GAP^b^	Cluster 6
kbo Kliniken Agatharied und Landsberg am Lech^b^	Cluster 7
kbo Klinik Taufkirchen/Vils^b^	Cluster 8
kbo München Ost Haar^b^	Cluster 9
kbo Kliniken München Nord^b^ and FFB^b^	Cluster 10
BKH Günzburg, University of Ulm^ab^	Cluster 11
BKH Kempten^b^	Cluster 12
BKH Kaufbeuren^b^	Cluster 13
BKH Memmingen^b^ und BKH Donauwörth^b^	Cluster 14

*kbo* Klinikverbund Oberbayern, *BKH* Bezirkskrankenhaus, *GAP* Garmisch-Patenkirchen, *FFB* Fürsenfeldbruck

^a^University hospitals

^b^Community hospitals

### Outcomes

#### Primary outcome

As primary outcome, guideline conformity among health care providers will be assessed by means of a ‘Questionnaire on guideline knowledge of the S3 Guideline Schizophrenia.’ As a subjective notion of guideline compliance does often not depict reality, the primary outcome is based on case studies developed for this purpose. The questionnaire is developed in correspondence to a publication in the field of internal medicine investigating guideline compliance [25]. For this purpose, 46 casuistic, i.e., case vignette oriented multiple choice questions are asked and a quantitative (number of appropriate answers in relation to the guideline) as well as a qualitative aspect (five cardinal questions were identified as being most important by the investigators and were selected from the 46 questions) will be investigated. Thirty out of 46 items including five correctly answered cardinal questions will be operationalized as guideline compliant and will serve as an evaluation of the effectiveness of the implementation strategies. If less than five cardinal questions were answered correctly, the participant fails, regardless of the number of correct answers. This additionally serves the evaluation of the efficacy of guideline implementation. Differences within the descriptions of the case studies and different sequence of the questions are used for the time points T0 and T1 in order to avoid learning effects.

#### Secondary outcomes

The following outcomes (adapted for the trial purpose where necessary) are collected as secondary outcome measures:

- Digital health expertise measured with the eHealth literacy scale [26]
- Usability of the respective formats by the ‘System Usability Scale’ [27]
- Caregivers’ confidence in decision-making measured via the ‘Provider Decision Process Assessment Instrument’ as well as the ability in shared decision making using the ‘Participatory Decision Making Questionnaire’ (PEF-FB-Doc) [28, 29]
- Furthermore, attitudes towards and use of the respective formats (traditional print format or MAGICapp) will be recorded.

### Data collection and management

During the examination statements and personal information of the participants is gathered and electronically stored. They will be stored in double pseudonomized form, no names or initials will be used but a code with numbers and letters. In the data table there will be no entry who filled in the data, when and from where the survey was filled in. The software LimeSurvey ® fulfills the German valid data protection standard and the complete process was approved by the responsible data protection officer of the LMU university hospital. The saved data will be stored according to national regulations in the archives of the Department of Psychiatry, Psychotherapy, and Psychosomatics of the University of Augsburg. The data is provided on a voluntary basis after giving consent. Participants are informed about the basis regulation of data protection (Art. 12 ff. DSGVO). Furthermore, they may withdraw their consent at any time.

### Sample size justification and planned data analysis strategy

Sample size is based on the primary outcome. Our calculations are based on the results of the already conducted survey, which has shown only 40% orientating to the guideline in clinical practice [10]. We therefore conclude p1 = 0.4 of the participants in the control group will reach the cut-off regarding guideline compliance (≥ 30 points and five correctly answered cardinal questions). In the experimental group (MAGICapp), we expect p2 = 0.6, meaning that in this group, 50% more than in the control group will reach the cut-off to guideline compliance. Applying the two-sided Fisher’s test with *α* = 0.05, a power of 1-*β* = 0.8, and a distribution of N2/N1 = 1, it is calculated that 204 participants were required if no cluster randomization was applied [30]. In this cluster randomized trial, the design effect (DE) is needed to calculate the final sample size [31]. The DE is defined as DE = 1 + (*m* - 1) * *ICC*, with *m* being the average cluster size and *ICC* the intracluster correlation coefficient. We assume an average cluster size of 17 and an *ICC* of 0.01 [32] leading to correction factor of 1.164 (DE = 1 + (17 − 1) * 0.01). Consequently, the sample size is *N* = 204 * 1.16 = 237 participants, which means on average 237/14 = 16.93 per cluster.

### Planned data analysis strategy

For the actual survey, descriptive statistics will be calculated for each center as well as center-overlapping. Descriptive data of drop-outs and completers will be compared. For the primary outcome, a confirmatory hypothesis test will be performed with a two-sided significance level set to 5%. The analysis (Fisher’s exact test) will be based on an intention-to-treat population, which means that all clusters/participants are analyzed as randomized. Drop-out will conservatively be defined as non-compliant guideline adherence. However, we assume a minimal drop-out rate of ≤ 10%, as we examine care providers in two defined service regions. For secondary outcomes with continuous variables, linear mixed model will be applied with inclusion of data with missing values. If normal distribution assumption is violated, monotonous and continuous data transformation (logarithmic or inverse normal transformed (RIN, e.g., rankit)) or non-parametric tests (Mann-Whitney *U* test, Wilcoxon test)) will be used. The per-protocol population will be analyzed in a second step and will be defined as all participants with available T0 and T1 data for the primary outcome measure.

### Trial status

The current protocol version is 1.3 (12.02.2022). The date of first enrolment was 16 May 2022 and is completed due to the design of the study as a cluster-randomized trial. The estimated date of the end of data recruitment will be 30. November 2022.

### Ancillary and post-trial care

There is no anticipated harm and compensation for trial participation. Within all participants, 50 specialist books are raffled as compensation of the time spent for the study.

### Dissemination

The results of this protocol study will be published in peer-reviewed journals and presented at national and international conferences. All project partners agreed on a publication and authorship agreement, which regulated the procedure for publications in detail.

## Discussion

The purpose of guidelines is to translate research and its results into clinical practice and support clinicians in their everyday decisions. However, the implementation of guidelines in clinical practice is despite long years of implantation research insufficient up to date. This gap keeps persisting if implementation is not regarded in a given context—the success of complex interventions is based on an integration of both implementation and context [33]. The process of integration is described by the ‘Context and Implementation of Complex Interventions (CICI) framework’ [33]. This framework comprises three with one another connected dimensions—context (seven domains—geographical, legal, political, epidemiological, socio-cultural, socio-economic, ethical context), implementation (five domains—implementation theory, process, strategies, agents and outcomes), and setting. The setting is the physical location, in which the intervention and its implementation interact with the context.

A variety of different implementation strategies exist [6, 7]. There is probably no superior strategy fitting to all situations [4, 11, 34]. Moreover, insufficient guideline adherence indicates that no implementation strategy has sustainably succeeded so far. Consent has been reached that guideline implementation can be achieved (1) through multifaceted intervention strategies [7, 8, 35–37], (2) the identification of barriers and facilitators [12, 34], and (3) an involvement of the guideline users in the guideline development process [12, 35].

The multifaceted strategy of the SISYPHOS project includes different aspects of the CICI framework and aims at closing the gap between research and practice as well as leading to a behavioral change concerning guideline adherence. The results of the cluster-randomized study will be used for the update of the German schizophrenia guideline as the worldwide first living schizophrenia guideline. This trial is the first trial evaluating the efficacy of a guideline implementation with the evidence ecosystem MAGICapp following the idea to overcome the evidence-practice gap [4]. However, one meta-analysis indicates that classical implementation strategies of non-living guidelines, such as distribution of educational material, educational meetings, reminders, or feedback, have limited impact of the provider performance [4]. In that regard, it has to be investigated whether novel guideline formats (e.g., living guidelines) presented in an evidence ecosystem have an added value in provider performance—this concept will be investigated in our trial. A schizophrenia living guideline will make current evidence-based knowledge for treatment of patients with schizophrenia anytime available. In this context, a sustainable implementation of the guideline for schizophrenia in a living guideline format could be achieved. In summary, until now, it remains elusive whether living guideline systems are superior in terms of the user’s knowledge gain. This question will be answered with the planned trial.

## Supplementary Information

**Additional file 1. **SPIRIT Checklist for *Trials*.

## Data Availability

The datasets generated and/or analyzed during the current study are available from the corresponding author on reasonable request after publication of the primary endpoint.

## Abbreviations

AWMF: Working Group of the Scientific Medical Societies
CICI: Context and Implementation of Complex Interventions
DE: Design effect
DGPPN: German Association for Psychiatry, Psychotherapy and Psychosomatics
DRKS: German Clinical Trials Register
GRADE: Grading of Recommendations, Assessment, Development and Evaluation
ICC: Intracluster correlation coefficient
ICTRP: International Clinical Trials Registry Platform
LMU Munich: Ludwig-Maximilians-Universität Munich
MAGIC: Making GRADE the irresistible choice
PEF-FB-Doc: Participatory Decision Making Questionnaire
SISYPHOS: Structured implementation of digital, systematically updated guideline recommendations for enhanced adherence in schizophrenia
